# The ‘Myth of Zero-COVID’ Nation: A Digital Ethnography of Expats’ Survival Amid Shanghai Lockdown during the Omicron Variant Outbreak

**DOI:** 10.3390/ijerph19159047

**Published:** 2022-07-25

**Authors:** Benjamin H. Nam, Hans-Jörg Luitgar Weber, Yuanyuan Liu, Alexander Scott English

**Affiliations:** 1 School of Education, Shanghai International Studies University, Shanghai 201613, China; w2004@shisu.edu.cn (B.H.N.); hjlw@shisu.edu.cn (H.-J.L.W.); liuyuanyuan@shisu.edu.cn (Y.L.); 2 Department of Psychology and Behavioral Sciences, Zhejiang University, Hangzhou 310027, China

**Keywords:** global migration, resilience, secondary coping, social media, COVID-19

## Abstract

This study presents a digital ethnography of expats’ survival amid the Shanghai lockdown during the Omicron variant outbreak. This study drew insights from studies on resilience and secondary coping within the context of global migration to comprehend the diverse emotional challenges faced by expats in a series of lockdowns and persistent nucleic acid amplification tests. Thus, this study asks what the major emotional challenges expats faced and what sources of social support they could draw from citizens in their host country during the Shanghai lockdown. Accordingly, this study collected WeChat group conversations to draw empirical findings, promoted scholarly conversations about fundamental survival necessity, and traced the process for establishing intercultural collective resilience with citizens from their host country. Overall, this study emphasized the significance of host country members who can promote certain coping mechanisms for their visitors in the specific regional and geographical context of China.

## 1. Introduction

Shanghai is one of the world’s most influential cosmopolitan cities, attracting numerous expatriates to settle down and contribute to China’s national economy as well as to the world economy [1,2]. However, the recent outbreak of the Omicron variant hampered these foreigners from pursuing their social and emotional wellbeing. In retrospect, China normalized the level of the pandemic issues in the early days of the COVID-19 pandemic through its “Zero-COVID” policy, in which the government strictly controlled internal transportation and cross-border traffic [2,3,4]. Notably, when the Delta variant spread in China during the summer and fall of 2021, the government implemented a series of citywide lockdowns in key global economic hubs in the Yangtze River Delta region and Southeast China (e.g., Hangzhou, Nanjing, Suzhou, and Shenzhen). In so doing, the nation quickly normalized the public health crisis [5]. Indeed, Shanghai’s precision prevention model has been valued by Chinese society as a whole, as the city implemented certain strategies to control explosive chains of lethal transmission. Shanghai residents showed strong collective behaviors by wearing face masks regardless of age, gender, occupation, or location in looking out for their neighbors’ wellbeing [1].

Despite these efforts, the recent outbreak of Omicron in Shanghai has led to a long-term citywide lockdown. This lockdown has been a frequent topic in global discourse, as Shanghai is one of the most economically advanced cities in the global arena, playing host to numerous foreigners. According to mainstream global public media outlets (e.g., American Broadcasting Company, British Broadcasting Company, Global Times, and South China Morning Post), many nations have adopted strategies for living with the coronavirus as the consistently growing infection rate seems to have made it impossible to return to zero cases. However, China has continued its “Zero-COVID” policy through the government’s strong social control [6,7]. Since 2 March 2022, more than 28.5 million residents including approximately 160,000 foreigners have endured a series of short-term (48 h) and long-term (14 days or more) lockdowns and repeated Polymerase Chain Reaction (PCR) tests [8,9]. Notably, Shanghai had a relatively high level of vaccination rate, which showed approximately 85% of all residents were vaccinated by the time of the Omicron outbreak. However, while approximately 62% of senior citizens were vaccinated, only about 38% of them received booster vaccinations [10]. In this rapidly growing domestic problem, the emotional and social well-being of numerous expats under the strict ‘Zero-COVID’ policy has also been an issue of increasing geopolitical concern for foreign embassies, consulates, and chambers of commerce in Shanghai. Foreign nationals have felt an enormous amount of uncertainty, confusion, stress, and frustration [11,12].

Although there is a growing body of literature addressing the impact of the COVID-19 pandemic on the human ecological system as well as recent global public media coverage of the current Shanghai lockdown under the ‘Zero-COVID’ policy in China, scholars and journalists have paid scant attention to the lived experiences of residents, including expats, in the locked down city. At this point, it is significant to recognize that “explicit cross-cultural adaptation is already stressful and continues onward to contemplate how emotional factors interact with psychological trauma during the pandemic” and address how members of host nations could reduce cultural conflicts and promote social and emotional wellbeing for expats by establishing potential coping mechanisms [13].

Moreover, exploring the nexus between anxiety and coping during a lockdown can increase our understanding of the perceived impact and wrong information sources, which can accelerate the level of emotional unrest, especially social media effects on ordinary people [14]. It is also crucial to pay close attention to potential moral injury that could influence job burnout during a long-term pandemic time; medical professionals, healthcare providers, and community volunteers can face a moral dilemma, “when one is aware of the right thing to do but is unable to do so because of occupational constraints” [15]. From these perspectives, the current study expands on previous scholars’ claims about the importance of host nation citizens at a time when expats feel confused and uncertain about their life changes. This study applies a digital ethnographic approach to explore the social and emotional challenges faced by expats and to determine from a cross-cultural perspective the factors that boost intercultural collective resilience through secondary coping for expats.

## 2. Emotional Challenges Amid COVID-19 Lockdowns in the Chinese National Context

Emotional challenges are defined as traumatic psychological symptoms that include stress, confusion, frustration, depression, and the feeling of loss. Due to COVID-19 lockdowns, numerous individuals worldwide have been facing diverse forms of emotional challenges [13,16,17]. A growing body of literature has explored the emotional challenges faced by people under COVID-19 lockdowns. Previous scholarship investigated Wuhan’s 76-day lockdown (23 January–8 April 2020). For example, the long-term lockdown significantly impacted Wuhan residents’ emotions and empathy, creating anxiety about physical and mental health. The most severe traumatic incidences and risk factors were lack of necessities such as food, drink, and medical supplies (e.g., mask and emergency medicine) [13]. Another growing concern was the limited number of medical staff could not cope with the rapidly growing case rate. Phobia and stigmatization of Wuhan residents were also persistent concerns as the city had the most infections (67,733 out of 80,955) in the country [13,18,19].

In global migration research, previous scholars explored the pandemic experiences of educational sojourners (e.g., international students and faculty) including Chinese international students residing in foreign countries and international students in China. For example, Nam and his colleagues [20] examined racially traumatic events and potential risk factors among 16 Chinese international students and eight Chinese exchange students during the early days of the COVID-19 pandemic (spring semester of 2020) at U.S. institutions of higher education in Arizona, California, Florida, Massachusetts, New York, Oregon, and Texas. While schools were closed, often Chinese students were in quarantine or self-imposed isolation due to growing Xenophobia and Sinophobia. They were also anxious about consistent public and social media portraits that fueled the stigmatization of ethnic Asian people, especially positioning Chinese people as potential vectors for coronavirus infection. Due to racially traumatic events and their security concerns, many of the participants who were on educational exchange (17 out of 24) returned to China after the 2020 spring semester [3,20].

Additionally, another study investigated the emotional challenges faced by eight international students from Pakistan, Malawi, and Cameroon who were unable to be evacuated during the Wuhan lockdown. Their symptoms consisted of homesickness, stress, anxiety, and fear. Although they received social and emotional support from school administrators, embassies, and locals, they testified that they still had long-traumatic symptoms [13]. Finally, the other studies explored the initial nationwide lockdown experiences of international faculty in Shanghai and graduate student researchers in Beijing with which the timeframe when Wuhan residents were in a long-term lockdown. They faced extreme stress dealing with online teaching and learning as well as intercultural communication gaps. They also witnessed many individuals’ mental crises regardless of national origin, social representation, or cultural practice [2,4].

## 3. Theoretical Framework

### 3.1. Resilience in Cross-Cultural Psychology

A growing body of literature within cross-cultural psychology and in the broader field of social and behavioral science has sought to comprehend migrants’ coping mechanisms for handling emotional challenges [17,21,22,23,24,25]. Resilience is one of the considerations to which COVID-19 researchers recently have paid more attention in order to explore the factors that impact individuals’ mental crises and the challenges to overcome diverse emotional barriers [17,21,22,23,24,25]. Explained simply, when it comes to the pandemic, the fundamental concept of resilience illustrates “the rate of recovery of a system from perturbation back towards a presumed, pre-existing stabler state–here zero infection and associated deaths–where rapid recovery equals high resilience” [24].

In the field of cross-cultural psychology that deals with global migration, scholars often consider the concept of resilience when viewing the acculturative challenges faced by sojourning groups. Simply put, acculturation is defined as “the general processes and outcomes (both cultural and psychological) of intercultural contact” [26,27]. There are four types of acculturation patterns in plural societies: (a) integration; (b) assimilation; (c) separation; and (d) marginalization. These four patterns are considered to be the consequences of immigrants’ attitudes toward adopting a new cultural identity in their host society or maintaining their ancestral cultural identity. A positive attitude toward both maintaining one’s original culture and accepting other cultures yields integration. Notably, resilience is to identify the moderating factors that promote coping mechanisms for migrants who face emotional challenges by examining the ways in which host country members can serve as moderators and help to bridge the cultural distance between the host society and the expats’ culture of origin [27].

In underpinning the concepts of resilience in COVID-19 research, resilience is a form of community relationship and social interaction among members of specific inner cultural groups or selective cultural groups [28]. For instance, Wuhan residents used prosocial behaviors to support one another, using social media apps to seek volunteer groups (e.g., medical supplies and food deliveries) at the local level. Controlling social deviance or cultural conflict is a crucial factor that boosts resilience, helping cultivate prosocial behaviors and a sense of belonging amid lockdowns [13,18]. Additionally, prosocial behaviors among members of inner cultural or selective groups can influence other cultural groups, motivating them to cultivate compassion and humanitarianism and to help their neighbors in times of public health crisis [21].

### 3.2. Secondary Coping

Secondary coping illustrates how sojourners and migrants deal with stress and anxiety through creating collective endeavors to overcome cultural conflicts. This type of coping indicates that sojourners and migrants are negotiating with their emotional challenges, developing mindfulness and willingness to accept certain negative events or memories rather than denying or committing self-imposed isolation [29,30]. Cultural conflicts or adaptation challenges can occur because of intercultural communication gaps such as language barriers and misunderstanding of local cultures [31]. The importance of social and emotional support from local residents as a prominent coping strategy. For instance, prior to the COVID-19 pandemic, migrant workers and international students faced cross-cultural adaptation challenges due to language barriers and social values in China due to host nation members’ strong emphasis on collectivist identity. Yet, they felt less pressure when they were socially and emotionally supported by the local community [32,33].

Additionally, using secondary coping (e.g., emotional and social support) for international students amid the Wuhan lockdown decreased their emotional challenges and helped to create a positive image of the host nation despite ongoing psychological traumatic symptoms such as stress and anxiety about physical and mental health, and homesickness and burnout from studying abroad. Social support from university administrators and Chinese friends helped them to regain their motivation to undertake their academic studies [13].

Pertinent to the current study, it is necessary to understand Chinese collectivism because of its preference for tightly knit social networks in which individuals can expect their relatives, friends, and/or communities to look out for them in exchange for unquestioning loyalty. This worldview has been linked to historical farming cultures in China. The rice farming cultural region left a lasting influence on southern China, which has implications for millions of people who are descended from rice-farming communities. In this context, rice farming’s intense labor demands led to cooperative labor exchanges, and rice irrigation networks led to social coordination, monitoring, and punishment systems. Although very few people in Shanghai farm rice today, this history led to tighter social norms and more stable social relationships [34].

### 3.3. The Rationale for the Current Study and Research Questions

Despite the fact that the past two years have witnessed the emotional and social well-being issues involving numerous individuals during the COVID-19 pandemic, the experiences of expats amid the long-term Shanghai lockdown along with the new Omicron variant have been paid limited attention. When most countries have increased their cross-border traffic and normalized the level of the pandemic issues, China has been continuing its dynamic Zero-COVID policy. In this context, expats in China could be vulnerable populations based on language barriers and cultural distance [33]. Notably, expats’ experiences of the consistent short-term and long-term lockdowns in Shanghai (2 March–1 June) can significantly be different from other previous lockdown cases including Wuhan’s 76-day lockdown, because of frequent policy changes, food and water shortage, fake news, and lack of emotional and social support from their own diplomatic communities, among others but not because of the unknown virus and its explosive chains of lethal transmission. Based on the chosen theoretical considerations, assumptions, and contexts, the primary research questions that guided this study were:

RQ1: What were the major emotional challenges faced by expats during the series of lockdowns to contain the Omicron variant outbreak in Shanghai?

RQ2: What sources of social support did expats receive from host country members to cope with their emotional challenges?

## 4. Materials and Methods

### 4.1. A Digital Ethnographic Approach

We, the four authors of this study, are transnational researchers: one Asian-American man, one White-American man, one German man, and one Chinese woman. We are conventionally trained academics in comparative and international education, cross-cultural psychology, cultural geography, and sociolinguistics. We met through professional relationships in an institutional setting as academic faculty members affiliated with an institution of higher education in Shanghai, China, and three are expats with high-level foreign talent visa status in teaching and research. All of us have been enduring the COVID-19 pandemic in China since the initial outbreak. Three of us have now experienced a series of lockdowns and persistent PCR tests in Shanghai since 2 March. Yet, one of us, a foreign faculty member, recently moved to another institution in Hangzhou, which is about 150 km away from Shanghai, before the Omicron outbreak in China.

To implement this research, we adopted a digital ethnographic approach which is beneficial for examining social and behavioral changes among divergent culture-sharing groups by using new technologies for social research [35,36]. These broadly consist of communications via public and social media platforms, videoclips, blogs, letters, emails, and more diverse qualitative sources visible online [36]. Thus, in conducting a digital ethnography, ethnographers immerse themselves in other culture-sharing groups, examining diverse social issues and cultural events as observers [35,36].

### 4.2. Fieldwork via WeChat

**Figure 1:**
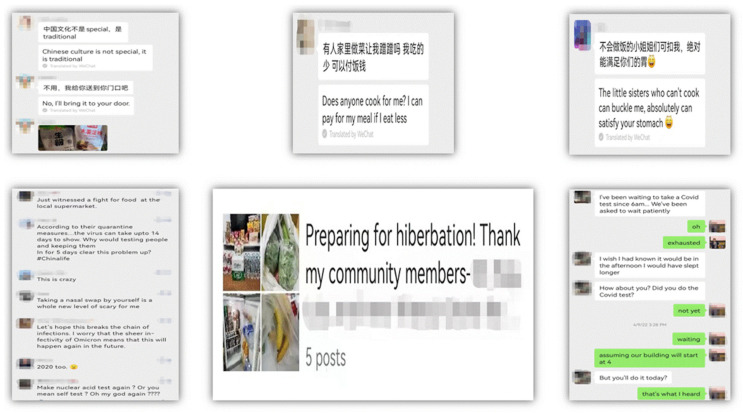
Example texts from WeChat group, moments, and personal communication.

We adopted an observational research approach [37]. Accordingly, we conducted direct and participant observations via WeChat, which is the most widely used social media messaging app in China. A total of 1558 WeChat accounts were divided into seven chat groups (see Table 1). We developed fieldnotes through our personal reflections, observations, and written and textual data such as media sources, email communications, and online meetings (e.g., Zoom and Tencent). These data sources were utilized to develop the contextual background for each theme. We also captured approximately 300 screenshots, which include but are not limited to sources of information from international diplomatic communities, compound committees, and volunteering-groups, as well as WeChat communications among various individuals. Some of the individual texts (personal communication) were also collected from those individuals. We mutually discussed and selected the most appropriate screenshots. Given this, we excluded texts that are too political, inappropriate, or vulgar (see Figure 1) [38].

Although we relied on web-based objects such as WeChat group data by conducting observations, we respected ethical guidelines according to our IRB protocol. Overall, we acknowledged that respecting individuals’ personal identities should still be protected by researchers. Thus, we obtained consent from only those expats who provided their own texts or personal messages online. We used pseudonyms to protect their identities, removing their specific affiliations and age.

### 4.3. Data Analysis

We used a qualitative thematic analysis method to deduce conclusive findings [39]. In the initial phase, we carefully reviewed texts in each WeChat group and mutually discussed to consider potential themes in relation to the primary research questions. We openly coded both common and diverging perceptions of the emotional challenges faced by expat groups. In the meantime, we considered mutual interactions between foreign expats and Chinese groups. In the next stage, we considered cross-cultural factors that promote or hamper expats from cultivating prosocial behaviors and collective resilience in inner cultural or selective cultural groups. We recognized that there were limited texts on prosocial behaviors or collective resilience among expat groups but found there were some positive aspects of collective intercultural resilience between the expat and Chinese groups. In the final stage, we mutually discussed the most influential and emergent themes to identify the emotional challenges faced by expats and the sources of social support from Chinese citizens.

## 5. Results

### 5.1. Expats’ Emotional Challenges Amid the Shanghai Lockdown

On 2 March 2022, two of us were teaching on campus. It was the first week of the new spring semester. That day, the Shanghai government announced that the first infection case had been found at the Flower Hotel in downtown Shanghai. All classes were immediately canceled, and all academic faculty, administrative staff, and students were required to stay on campus and take PCR tests. One of us returned home at night. One of us remained on campus for more than three days to serve as emergency staff to organize a series of PCR tests over the following days. Since that day, we have experienced multiple short-term (48 h) lockdowns and consistent PCR tests over the course of four weeks, before the major lockdowns were mandated on 28 March or 1 April. From our own personal standpoint, we experience neuroses about the omen of lockdowns. We were extremely exhausted and frustrated due to the persistent PCR tests. We were also stressed about our ordinary lifestyle shifts. We also witnessed many individuals express leisure constraints, which made them feel burnt out and disengaged from their jobs. We have also had concerns about families and friends, experiencing or witnessing separation, illness, loss, and grief during the series of short-term and long-term lockdowns affecting Shanghai (Fieldnote, 19 April 2022).

### 5.2. Fake News and Rumors about the Major Lockdown

On 28 March, the Chinese government implemented a citywide lockdown policy. The government announced that the eastern part of the city (浦东: Pudong area) would be sealed for four days from 28 March to 1 April and in turn, the western part of the city (浦西: Puxi area) would be sealed from 1 April to 5 April. Accordingly, over 28 million Shanghai residents were isolated into their compounds in the hope that the city could contain the outbreak of the Omicron variant (Fieldnote, 12 April 2022).

Expats were confused and uncertain due to consistent fake news and rumors about the major lockdown. They were already extremely stressed out and exhausted from the multiple short-term lockdowns. On 7 April, texts between members in the EU-Citizen-Group showed how confused they were about the rumors. Staying in different districts and compounds, they wished to collect accurate information in a moment when there were growing rumors about when their compounds would be unsealed:

Polish man: I heard from Chinese people. Some compounds will be unsealed soon. Anybody heard of it? ... Or other restrictions? I heard our compound is on the list!

German man: It’s still the same here. Nothing’s changed.

Polish man: I can hardly imagine that any shop is able to open.

French woman: If we go out, one person from each household will be allowed to go get some food per day.

In this group communication, they recalled their past lockdown experiences when the initial COVID-19 outbreak was growing into pandemic in late January through the middle of March 2020. We also remember that the Chinese government implemented a nationwide lockdown in response to the rapid rise in infections in Wuhan, so Shanghai residents were subjected to multiple lockdowns. Yet, their emotional challenges in the current major lockdown differed greatly from the Wuhan lockdown because in 2020 there was fear and uncertainty about an unusual pneumonia, while the 2022 Shanghai lockdown seems to be more about stress and anger due to the uncertain policy that was rapidly implemented and the rumors being spread (Fieldnote, 12 April).

Expats in the EU-Citizen-Group also discussed their confusion and uncertainty about the hope that they could go outside and purchase fundamental necessities. Here is the conversation:

French man: I guess, we will be partially allowed out, but only without motor vehicles.

British woman: Actually, we should be allowed to walk inside the compound tomorrow, but we haven’t heard anything yet. Let’s see.

German man: We also got two tests the day before yesterday, but no news yet. We’ll probably stay inside for another 14 days anyway. There’re still growing cases in our neighborhood.

Despite expats’ wishes, the long-term lockdowns have continued, though the government has partially unsealed compounds starting on April 11. To be unsealed, the entire compound must be free of infection for more than 14 days (Fieldnote, 13 April 2022). The expats’ emotional discomfort eventually became anger. For example, a British woman in the High-Level Foreign Talent-Group-1 expressed:

That actually makes me really angry when people just say everything’s going to be alright. Yes, there’s a lot that I’m grateful for, but there was a lot that could have been avoided because I honestly do believe there were people [the Chinese government] with inside information who knew this was going to last more than four or five days. They knew this, and that’s what makes me angry.

An American man in the High-Level Foreign Talent-Group-2 expressed similar sentiments: “I really believed that it would’ve been just four days, but my compound committee said, ‘we will have to stay home for a few more days’. I was confused about it, but it has just continued. Now, my faith’s gone”.

### 5.3. Food and Drink

As already noted, the current Shanghai lockdown is much different than previous lockdowns because all compounds were completely sealed from 28 March to 11 April. In previous short-term and long-term lockdowns, residents could order food and drink. However, they were not allowed to order these essential necessities. Moreover, as the number of infections has continued to grow, numerous compounds were sealed. Expats are exhausted physically and mentally, having self-PCR tested and reported it to their compound committees between midnight and 8:00 am (Fieldnote, 19 April 2022). Given this, the conversation between expats in High-Level Foreign Talent-Group-1 revealed the severe situation:

Canadian Man: So, I purchased enough food and stored up to five days. And then I realized the situation was not true. We filled our first trial, and yesterday we tried another order. But they say they will not deliver our order… They don’t know when they can deliver our order to us. So, at this moment, I still have a few packs of Macaroni in my stock. But other than that, I’m trying to get help from my neighbors… The greatest challenge to me this time was that I feel a lot of psychological pressure because whenever I turn on the computer and look on the Internet or sometimes watch TV, all the news is heartbreaking.

British woman: I am sure of this. I could have stocked up. There was food in my neighborhood. I could have stocked up on things I eat because what’s being offered to me. I don’t eat this stuff [Chinese food]. So, I could have stocked up on things that I eat. I could have had stuff to last me two months. But you know, I believe don’t panic and don’t be greedy, don’t hold. Just have enough for four or five days. So, this is what’s making me really angry. I feel really like so naive and so foolish. Like normally I’m too angry about this. Some people exploited this. Some people with inside information.

Korean man: I usually enjoy ramen. What I mean is Korean ramen. I went to a local Korean supermarket and bought some different sorts of ramen. I also bought some Korean cookies and drinks. I ate ramen for about a week straight and felt extremely nauseous. I thought it could be okay just for several days. I was worried about my health. I really do feel like I’m in jail.

Although food and drink were available as the government distributed them, or the expats’ companies delivered provisions, some expats expressed frustration regarding food and drink, especially the cultural practice that have their own choice of meal. They also discussed increasing food prices or pointed to difficulties in purchasing drinks such as wine or beer. Notably, for German people, beer and fruit are significant when they have meals as a cultural practice. For French and British people, cooking, baking, and drinking wine are also important as cultural practices when they have meals. Expats’ texts in the EU-Citizen-Group demonstrated these frustrations:

German woman-1: The price is crazy. 5510 RMB [Chinese currency] for a box of grapes?

German woman-2: My apology. It’s 560 RMB for 9.5 kg.

German man: Any kind of alcohol should be ordered or delivered.

French woman: By the way, I have beer. Do you have sugar? Please let me know if you want to exchange.

British woman: My husband ordered beer somewhere last week, but it hasn’t been delivered. It only lasts five minutes.

German man: At least, our embassy must solve these issues.

Expats in other groups also expressed cultural issues. For instance, a British woman in High-Level Foreign Talent-Group-1shared, “I can’t have three meals [of Chinese food]. I really can’t. Psychologically, I cannot. I need noodles that I usually enjoy.” A Turkish man in High-Level Foreign Talent-Group-1 who is a practicing Muslim also raised concerns about his food choice. He shared, “Actually, we have very specific cultural and religious backgrounds. That’s why we don’t prefer to eat pork. We are pleased to get some chicken or beef. These are basic needs for us. I hope I’ve made myself very clear”.

### 5.4. Family Wellbeing: Potential Separation from Children and Pets

On behalf of the EU member states (24 nations), the Consulate General of France in Shanghai represented their complements to the Foreign Affairs Office of the Shanghai Municipal People’s Government and drew their attention to the following matters. The EU member states demanded that under no circumstances should children be separated from their parents. For asymptomatic or mild infection, it was best to set up a special isolation environment and communicate with key staff in English. Notably, the EU member states emphasized the need for timely and effective access to emergency medical assistance when required by their citizens during lockdown (Fieldnote, 10 April 2022) (Consulate General of France in Shanghai shared the official documentation on 1 April 2022).

During the lockdown, expats were anxious and concerned about their family wellbeing, especially the potential for separation from their children and pets. From a cultural perspective, many foreigners consider pets to be family members. In our observations, there was growing concerns among expat groups with children and pets, and they posted about their emotional states and discomfort regarding potential separation issues that were consistently shared by their friends or civil entities. There was also increasing fake news and rumors regarding animal abuse (Fieldnote, 19 April 2022).

Concerning the potential for separation, a German man in the EU-Citizen-Group stated: “I heard that a foreign man was positive and just returned to his family, but he was told that he is positive again, so he was required to go to the camp or mobile hospital again. He has a little daughter and argues with the government officials and refuses to be in long-term quarantine again.” An American man in the U.S.-Citizen-Group also heard the story, whether it was a rumor or not, and stated, “The story was spread on social media for a while but is no longer found elsewhere…I presume [nationality], but not making any official word to that, references his foreign friend in [another city] who experienced something like this. So, we can obviously assume this is an accurate account.” A British woman in the High-Level Foreign Talent-Group-1 expressed, “They are taking away our kids from us! I’m not afraid of COVID infection but anxious about taking our kids. It’s not appropriate.” Likewise, expats who have children have been paying more attention to potential separation issues and feeling pressure to maintain their families’ health conditions.

With respect to potential separation from pets, for example, a post on WeChat moment shared by an American woman in the High-Level Foreign Talent-Group-1 read:

American Woman: Urgent help needed! Our shelter will be in lockdown! As you know, there is going to be a 5-day lockdown which means that nobody will be able to come and take out/feed or walk our little shelter dogs. I’m in an absolute stress about this because it means for 5 days our shelter will be empty and the dogs will all be sitting in cages (25 March 2022).

Additionally, on 6 April 2022, an article was shared widely on social media platforms such as WeChat moment and That’s Shanghai that described “a corgi [dog] was killed by a volunteer” right after “its owner’s COVID-19 test result was positive and prepared to go into isolation.” (Fieldnote, 6 April 2022) [40]. A conversation between two American men in the U.S.-Citizen-Group discussed this issue:

American Man-1: Crazy

American man-2: [Friend] posted it on her moment.

American man-2: It was exactly two years ago. I was jogging and a lady was walking her dog and used a stick to beat the dog in public.

American man-2: For us, pets are also family members.

American man-1: Yep. These guys are family to us.

American man-2: I also see Chinese people love pets. They walk their dogs and even hold cats outside while they’re walking.

American man-1: It’s a very stressful time. Those volunteers who take the infected away consistently. I can see they’re frustrated, but they have no such authority to kill animals.

Overall, expats with children and pets were concerned about the potential for separation, dealing with their emotions when they heard about events on social media, whether rumors or not. They acknowledged that it was extremely stressful for everyone. Yet, they argued that those with policy-making authority have no right to take their children or pets.

### 5.5. Social Support from Chinese Citizens

The international diplomatic communities shared their ideas to protect their citizens in Shanghai in the course of the Omicron outbreak. The EU member states emphasized the need for timely and effective access during the lockdowns to emergency medical assistance when their citizens required it. If citizens of EU member states and their families could enter Shanghai’s two international airports [Pudong and Hongqiao airports], the Chinese government should allow them to leave (Fieldnote, 10 April 2022). On 8 April 2022, the U.S. Consulate General in Shanghai urgently emailed and invited 500 citizens to attend an online meeting via Zoom. The consul general discussed a few key agendas including: (a) U.S. citizen services; (b) food and water; (c) child separation and infection treatment; and (d) evacuation plans (One of the American authors received an email from the U.S. Consulate General of Shanghai and attended a Zoom meeting).

Briefly, U.S. citizen services were temporarily closed on 30 March, but the Consulate General would resume regular services as soon as possible. Moreover, the Consulate General had been communicating with the Shanghai government and negotiating with them regarding the food and water as well as delivery services. Additionally, as the one of the major concerns involved the potential for child separation and infection treatments, the Consulate General would prioritize citizens’ safety issues. Notably, regarding the evacuation plan, there were commercial flights but no guarantee that every citizen could utilize them. Therefore, there would not have been U.S. government assistance in the event of an evacuation. However, the Consulate General would closely interact with the international diplomatic community, especially EU and French diplomats, to protest all fundamental requests (Fieldnote, 10 April 2022). The U.S. government recently implemented an executive order in which non-emergency consulate staff, diplomats, and their families in Shanghai return to the U.S. prior to 11 April. Thus, their business and services have provisionally stopped (Fieldnote, 10 April 2022), [11].

Despite the international community’s announcement, expats have not been supported by their own embassies and have faced various emotional challenges. In our observations, many complained about their circumstances due to the lack of support from their own embassies and consulates, whether their countries were diplomatically advanced or not. Further, most conversations in WeChat groups did not demonstrate prosocial behaviors even in inner cultural or selective cultural groups. However, we found that Chinese people provided some positive social and emotional support and at times, and that they developed intercultural collective prosocial behaviors between expat and Chinese groups but not with their international diplomatic communities at large.

### 5.6. Survival Necessity Exchange

After 5 April 2022, many expats posted in their WeChat group chats or on their WeChat moments about dwindling supplies of food and drink as well as other fundamental necessities, posting pictures showing empty pantries or refrigerators. These issues were also common for Chinese residents. Yet, Chinese groups showed collective prosocial behaviors by sharing what they had. They left food or drink in front of their neighbors’ doors. For example, residents in the Chinese-Resident-Group expressed:

Chinese-1: Can anyone cook for me? I can pay for my meal.

Chinese-2: This time, you can’t even leave the house, and you can’t go downstairs.

Chinese-3: Notice, for those who can’t cook, I can cook for you and satisfy your stomach.

Chinese-4: Do you mind if I spare some? I’ll put something to drink in front of your doors.

Expats also engaged with these activities, sharing what they had with their neighbors. For instance, two expats’ communications with Chinese people in Volunteer-Group-2 showed:

American-1: I have over sixty cans of soda. I’m willing to share these. Also, for parents who have children, I have a bunch of cookies. I can deliver these items to you.

Chinese-1: Thank you. Would you like some beer?

Turkish man: Anyone who wants to try Arabic coffee? I’ll offer free coffee.

Chinese-2: Checked the message now. Is it still available?

After 11 April, many Shanghai residents were able to purchase food online. Since expat and Chinese groups have been building international relationships through prosocial behaviors during the major lockdown period, sharing food and drink has become a common cultural practice, promoting social and behavioral changes in positive ways regardless of national origin, race, or ethnicity.

### 5.7. Emergency Volunteer Groups from Compound Committees and Companies

For expat groups, volunteer groups were formed from their compound committees and from their companies. Due to the lack of delivery services, expats’ companies, including educational institutions, could not support their foreign employees. Thus, compound committees formed volunteer groups to support these expats. Individual residents also volunteered to support them. Volunteer groups were composed of fluent English speakers. They generally supported delivery services. They also checked PCR test schedules for the expats. Key administrators or personnel staff members from their companies also joined the voluntary WeChat group, closely interacting with their residents. For example, a conversation in Volunteer-Group-1 demonstrated a form of volunteerism:

Chinese Personnel: We are sending food packages to you. We need your home addresses and cellphone numbers. Please make sure that your phone is working fine, so that your compound committee can send the food package to you.

British Woman: I’ll let the volunteer know. Thank you. I don’t speak Chinese, but she does, and she needs to arrange delivery.

Chinese Personnel: That sounds like a good idea. Please give me her phone number.

Additionally, another volunteer group also showed similar types of service, consistently checking on fundamental survival necessities. For instance, members in Volunteer-Group-2 showed a certain volunteerism:

Chinese Personnel: We will contact the governmental office in your neighborhood and try to send them to you.

Iraqi woman: Hi, I need basic supplies like vegetables, eggs, and milk.

Turkish man: The same supplies to me as well.

Chinese personnel: Okay. I’ll give the list to our boss.

Iraqi woman: All thanks to those who contributed to sending these foods.

Turkish man: Thanks for the provisions you sent us.

The Shanghai government also sent food and drink to their residents. In this regard, volunteers in compounds informed them about specific items to be delivered to expats. Overall, while numerous expats were facing emotional challenges without fruitful support from their own embassies or their families and friends in their native countries, the local government and its authorities, employers, and neighbors in Shanghai supported them in a wide variety of ways.

## 6. Discussion

Despite the various emotional challenges, we found that expat groups actively sought to develop coping mechanisms, attempting to find sources of social support from host country members. All these elements can boost resilience and help reduce emotional challenges. From a cross-cultural perspective, we perceive that resilience is a form of collective behavior, respecting or learning about the cultural values shared by locals. The form of behavior can affect the progress of developing coping mechanisms such as denial, negotiation, and acceptance step by step. Some of the expats actively engaged with collective resilience among their neighbors and learned how to cultivate prosocial behaviors [13]. Previous research showed that expats often deny their emotional challenges and hesitate to communicate with locals, especially when their cultural norms differ significantly from the host country’s culture [29]. Yet, the fundamental message for expats was that once they negotiate or accept the challenging issues (e.g., lockdowns, food, and water), host country members can take on mediator roles to help promote social and behavioral changes, adjusting to a new culture where they are settling in and thereby overcoming social and emotional barriers [17,25].

Chinese collectivism is unique in that resilience is the idea of the nexus between families and societies, which not only shows the social importance of human-to-human connection but also the progression of sustaining safety [1]. Shanghai is in the Yangtze River Delta, which historically has been a rice farming region with an agricultural legacy spanning 5000 years of Chinese history. Scholars have long conceptualized the cultural differences between the historical rice farming society in China and foreign societies and have faced the challenges foreigners face in adjusting to the local society, especially its historical collectivist cultural patterns. Even though Shanghai is China’s most economically advanced city with one of the nation’s largest populations, its local culture originated in the richest rice paddy farming in the country. Thus, in this particular geographical and cultural context, the cultural legacy of rice farming on Shanghai’s collectivism could promote resilience and coping mechanisms, helping to heal expats’ emotional challenges or mental crises [1,35].

In a broader global context, the largest international and cultural groups come from North America and Europe, which values Western capitalism and individualism and focuses on the stressors that affect the individual and personal life. Meanwhile, local Shanghai collective culture focuses more on their society, connection, safety, and emotional management. This factor could differ greatly from other Asian countries such as Japan and Korea. Scholars recently raised critical questions about the societal changes and cultural shifts in Japan and Korea as they become more neoliberal and individualist societies [13,41,42]. From these standpoints, expats and their Chinese neighbors have different cultural values and social practices. However, some expats created positive attitudes, making efforts to communicate with locals in order to develop a general sense of connection. In so doing, they could develop coping mechanisms such as sense of belonging, perseverance, and resistance to risk factors (hunger and safety), when they could not receive support from their embassies.

With respect to secondary coping, it is important to recognize that expats’ language barriers and lack of cultural knowledge could have been minimized when they were interacting with locals and enduring difficulties together, especially the impact of the “Zero-COVID” policy that mandated a series of lockdowns under the government’s strong social control. While expats were under pressure due to potential separation from families, residents in Shanghai could feel empathy for the expats as a vulnerable population. Family separation could happen to anyone during this timeframe. Yet, when they rely on each other, they can increase mutual international understanding and intercultural awareness, seeking relief through avoidance, acceptance, or cognitive reframing (i.e., secondary coping). It has been found that coping preferences change according to the cultural practices through which individuals experience immersion in different cultures [29]. Frustration with unexpected situations increases the degree of anxiety. The pandemic life during a lockdown can be seen as an uncontrollable stressor, and secondary coping (e.g., acceptance or cognitive reframing) could provide relief [14]. These types of stressors could also be due to the cultural distance that may have increased acculturative stress, which may lead to greater difficulties in acculturating successfully [13,43].

Finally, we found an enormous amount of frustration, stress, and other emotional challenges among expats living through the Shanghai lockdowns. They were happy to live in the city and experienced a high quality of life, even during the COVID-19 era. Hence, their emotional challenges amid the Shanghai lockdown were much different than the Wuhan lockdown or citywide lockdowns during previous variant outbreaks because of the frequent policy changes. In this regard, people could not prepare enough survival necessities [6]. However, we also identified certain forms of coping mechanisms among residents in Shanghai regardless of nationality, ethnicity, or race: individuals provided more inclusion and social support in the time of public health crisis. From this standpoint, scholars should explore positive influences rather than challenges in COVID-19 research, thereby promoting collective resilience that establishes intimacy, social bonds, equity, inclusion, and prosocial behaviors [37].

## 7. Implications, Limitations, and Future Directions

There are a few practical implications. Initially, we addressed severe emotional challenges among expats amid the Shanghai lockdowns and potential opportunities to establish coping mechanisms. Notably, concerning the use of intercultural collective resilience, secondary coping especially can bridge intercultural communication gaps between expats and host nation members in a public emergency. As we have seen, foreigners witnessed prosocial behaviors among host country members and showed a willingness to accept local cultural practices. These positive aspects of coping mechanisms could increase cultural awareness, drawing social support from locals. Thus, we suggest cultural coaches, trainers, counselors, and consultants consider how their clients can develop mindfulness and appreciate differences among people, which will aid them in overcoming various barriers as committed members of a contemporary global society.

Furthermore, over the span of two years and counting of the COVID-19 pandemic, Shanghai’s precision prevention model was highly regarded by the entire Chinese society. Accordingly, expats enjoyed their social and cultural life as well as leisure activities, contributing to promoting consumption culture. However, they have gone through multiple lockdowns over the course of three months (as of 1 June 2022), feeling extreme stress, exhaustion, and burnout. A recent online survey conducted in Shanghai revealed that of the 950 expats who responded regarding their future in China, 85% would rethink their stay in China, 48% plan to leave as soon as the city is unsealed, and 37% may stay if the dynamic Zero-COVID policy is eased [44]. Hosting expats is crucial for Shanghai as a global financial hub because they significantly contribute to boosting the local and regional economy as well as the national economy (e.g., business, finance, and banking [44].

Expats have also dedicated themselves to reinforcing the infrastructures for science, technology, engineering, mathematics, information and communication technology, vocational education and training, and linguistic diversity (e.g., global talent scholars such as university academics, researchers, and language teachers) [2,20,45,46]. Thus, it is significant to muse on Shanghai’s currently deteriorating image as a prominent cosmopolitan city. Key policy decision-makers in public health, foreign affairs, and urban planning should make a collective effort to reform appropriate policies and practices, thereby reestablishing the city’s positive image. In the meantime, they need to develop retention strategies for expats by providing essential alternatives so that they decide to stay.

There are a few notable limitations. At the time of writing, the Shanghai lockdowns are still ongoing. While we focused solely on the case of expats’ survival, studies of host country members’ experiences have so far been limited. We found how volunteer groups were urgently formed to support expats. It would be meaningful to explore how their collective identity was expressed while coping with the numerous structural problems of the “Zero-COVID” policy. Hence, we suggest future scholars consider how Shanghai residents dealt with their emotional challenges and established coping mechanisms in a time of public health crisis. Moreover, it is also instrumental to investigate how business leaders from global companies with local branches in Shanghai or other key economic cities in China foresee the dynamic of the ‘Zero-COVID’ policy. Future scholars should explore their perceptions about the prospects for both the Chinese and world economy when Shanghai is locked down. Furthermore, although this study used a digital ethnographic approach by observing 1558 WeChat accounts divided into seven chat groups and selecting texts upon the participants’ consent, the number of subjects was limited. Hence, we recommend that future scholars should conduct a quantitative study to support the current study’s findings and attempt to increase a better understanding of the concepts of resilience and secondary coping amid a long-term lockdown.

Additionally, during the time of the COVID-19 pandemic, numerous medical staff and healthcare providers have been making collective efforts to support patients and normalize the explosive chain of lethal transmission. Despite their endeavors, they often face job burnout and moral injury that could harm their professionalism such as righteousness, knowledge, and ethical behaviors upon dominant ideology and social norms [15]. Our study identified the adaptive nature of humans, even with different cultural values to survive and overcome the unexpected socio-ecological system crisis. In this regard, volunteer group members from the host country (i.e., Chinese) developed social and emotional support networks. Yet, along with a growing concern about the dynamic Zero-COVID-19 policy in China, it is worthwhile to investigate their moral injury and burnout factors. Finally, as numerous expats have been through a series of lockdowns in Shanghai without support from their international diplomatic communities, it is worth exploring how global public media platforms have portrayed lockdown issues. Future scholars should concentrate on how journalists and columnists viewed the myth of the “Zero-COVID” policy.

## 8. Conclusions

This study used a digital ethnographic approach to explore how expats experienced the Shanghai lockdown due to the spread of the Omicron variant outbreak. Our findings underlined the emotional challenges faced by diverse expat groups and the challenges they faced in seeking out coping mechanisms as well as gaining social support from host country members. Particularly, our study highlighted how those who may have had a positive image of Shanghai felt stress, frustration, and anger. Yet, our study also illuminated how their Chinese neighbors and employers served them as prominent mediators, establishing urgent volunteer groups to provide necessities. It is crucial to provide close attention to those vulnerable populations who face certain social and cultural barriers when compared to host country members. Accordingly, we suggested some notable practical implications for key policy decision-makers, business leaders, and a wider variety of stakeholders who can intervene into the visible structural problems of certain policies. Overall, we emphasize the significance of intercultural collective resilience in global migration research, especially when dealing with the emotional challenges faced by expats and the sources of social support in the specific regional and geographical contexts of Shanghai and China.

## Figures and Tables

**Table 1:** WeChat Group Information.

Group Pseudonym	Main Language	Number of People	Observation Period
U.S.-Citizen-Group	English	500	28 March–23 April
EU-Citizen-Group	English	489	2 March–23 April
High-Level Foreign Talent-Group-1	English	66	2 March–23 April
High-Level Foreign Talent-Group-2	English	265	2 March–23 April
Volunteer-Group-1	English	13	2 March–23 April
Voluteer-Group-2	English	6	8 April–23 April
Chinese-Resident-Group	Chinese	219	29 March–23 April

## Data Availability

Data is not publicly available due to anonymity concerns. Readers interested in the data can contact the first or corresponding author upon reasonable request.
